# Combining deep-inspiration breath hold and intensity-modulated radiotherapy for gastric mucosa-associated lymphoid tissue lymphoma: Dosimetric evaluation using comprehensive plan quality indices

**DOI:** 10.1186/s13014-019-1263-7

**Published:** 2019-04-08

**Authors:** Seo Hee Choi, So Hyun Park, Jason Joon Bock Lee, Jong Geol Baek, Jin Sung Kim, Hong In Yoon

**Affiliations:** 1 Department of Radiation Oncology, Yonsei Cancer Center, Yonsei University College of Medicine, 50-1 Yonsei-ro, Seodaemun-gu, Seoul, 03722 South Korea; 2 Department of Radiation Oncology, Jeju National University Hospital, Jeju University College of Medicine, Jeju, South Korea

**Keywords:** Mucosa-associated lymphoid tissue lymphoma, Radiotherapy, Planning study, Deep inspiration breath hold, Intensity modulated radiotherapy

## Abstract

**Background:**

Although there have been many attempts to increase the therapeutic ratio of radiotherapy for gastric mucosa-associated lymphoid tissue lymphoma (MALToma), only a few planning studies have reported the efficacy of the modern radiotherapy technique till date. Therefore, we performed the dosimetric comparison among 3-dimensional conformal radiotherapy (3D-CRT) and intensity-modulated radiotherapy (IMRT) plans, using deep-inspiration breath hold (DIBH) or free-breathing (FB) techniques, to determine the most optimal plan for gastric MALToma.

**Methods:**

We evaluated 9 patients with gastric MALToma for whom 3D-CRT, step-and-shoot IMRT (_S_IMRT), volumetric-modulated arc therapy (VMAT), and tomotherapy plans with identical prescribed doses were generated using DIBH or FB computed tomography (CT). Planning target volume (PTV) coverage and non-target doses were calculated for each plan and compared with plan quality metric (PQM) scores.

**Results:**

All 72 plans of 9 patients satisfied our dosimetric goals, and the IMRT plans and 3D-CRT plans had similarly good conformity index values with no differences related to respiratory movement. IMRT plans yielded significantly better doses to the organs-at-risk, and DIBH plans yielded significantly lower liver, heart, and lung D_mean_ and spinal cord D_max_ with smaller irradiated volumes compared to FB plans. For the mean PQM scores, VMAT-DIBH and _S_IMRT-DIBH yielded the best scores, whereas 3D plans provided reduced beam monitor unit values.

**Conclusion:**

Our findings demonstrate that modern RT technologies (DIBH with VMAT or _S_IMRT) could potentially provide excellent target coverage for gastric MALToma while reducing doses to organs-at-risk. However, the relevance of the most optimal plan considering clinical outcomes should be confirmed further in a larger patient cohort.

**Electronic supplementary material:**

The online version of this article (10.1186/s13014-019-1263-7) contains supplementary material, which is available to authorized users.

## Background

Mucosa-associated lymphoid tissue lymphoma (MALToma) accounts for approximately 19% of all non-Hodgkin lymphomas and can arise at any extranodal site. In Korea, however, at least half of all MALTomas present as primary gastric lymphomas [1, 2]. Several institutions have reported excellent disease control with radiation therapy alone, supporting the use of modest doses in field radiotherapy (30–40 Gy) for patients with stage I–II gastric MALToma [3–8], although systemic therapy may also be needed in cases involving unsuccessful *Helicobacter pylori* eradication or *H. pylori*-negative disease, depending on the disease stage.

The recent National Comprehensive Cancer Network (NCCN) guidelines recommend 30 Gy involved-site radiation therapy for gastric MALToma [9]; this generally involves the entire stomach and adjacent perigastric lymph nodes (if involved). Although this involved-site radiation therapy uses low doses, the doses to the organs-at-risk (OARs) near the stomach, such as the kidneys, small bowel, or liver, must be considered. To date, several planning techniques involving anterior-posterior/posterior-anterior fields (AP/PA) and 3-dimensional conformal radiotherapy (3D-CRT) have been used to reduce radiation exposure to the OARs to within tolerance limits, and intensity-modulated radiotherapy (IMRT) techniques for gastric MALToma have recently been introduced [10–15]. Despite the dosimetric advantages of these modalities in terms of the doses to the OARs, concerns regarding higher scattered doses have not been resolved [16]. Additionally, the effect of respiratory motion on the accuracy of the treatment has raised concerns, thus limiting the widespread use of IMRT.

Some institutions have implemented the deep-inspiration breath hold (DIBH) technique to address these concerns. However, only a few planning studies have included modern radiotherapy techniques when evaluating the efficacy of this technique [11–13, 17]. Therefore, in the present study, we performed a dosimetric comparison of 3D-CRT and IMRT plans using DIBH or free-breathing (FB) techniques to determine the most optimal treatment plan for gastric MALToma. We additionally compared IMRT plans using various techniques (step-and-shoot IMRT [_S_IMRT], volumetric-modulated arc therapy [VMAT], and tomotherapy) to identify the most dosimetrically optimal plan, using the plan quality metric (PQM) to ensure an objective assessment.

## Materials and methods

### Patient selection

Among 20 patients who received definitive radiotherapy for gastric MALToma at our institution between 2016 and 2017, we selected only patients who underwent computed tomography (CT) simulation scans using both FB and DIBH, to make 4 different plans (3D CRT, _S_IMRT, VMAT, and tomotherapy) per CT scan type (FB or DIBH) of each patient. A total of 9 patients who received definitive radiotherapy for localized gastric MALToma were selected consecutively for this planning study. All cases were either *H. pylori*-positive but unresponsive to *H. pylori* irradiation or *H. pylori*-negative. Before the start of radiotherapy, each patient underwent CT simulation scans (Aquilion LB; Toshiba Medical System, Tokyo, Japan) using both FB and DIBH, per our institutional protocol after at least 4 h of fasting. The patients received intravenous contrast agents and were immobilized in a supine position with both arms raised above the head. The range for CT scan was determined to include all the OARs (such as the lungs, heart, kidneys, and bowel) that should be considered in planning. In our institution, patients were scanned from approximately the level of the 7th thoracic vertebral body inferiorly to the level of the 4th lumbar vertebral body. Care was taken to include the base of the heart and both kidneys. DIBH CT scans were performed while the patient held his/her breath using the abdomen and chest motion self-control (Abches) system, as described in our previous report [18].

In all patients, the gross tumor volume (GTV) was defined as the whole stomach. However, different clinical target volumes (CTVs) were set for FB and DIBH CT, as the former must incorporate the concept of an internal target volume (ITV) to account for respiratory movement during treatment. The CTV was defined as the GTV plus a 1.5-cm margin for FB CT, and GTV plus a 1.0-cm margin for DIBH CT. To account for set-up errors, the planning target volume (PTV) was defined as the CTV plus a 0.5-cm margin for all CT scans. For all plans, the prescribed dose was equal to 30 Gy in 20 fractions.

Four different plans, including one 3D-CRT plan and three IMRT plans (_S_IMRT, VMAT, and tomotherapy), were generated per CT scan type (FB or DIBH) to yield 8 plans per case. Therefore, we compared 72 plans for 9 patients based on the planning modality and respiration control methods. This study was approved by the institutional review board (IRB) of the Yonsei University Health System (4–2017-1035).

### Planning techniques

The FB and DIBH CT images and all datasets were transferred to treatment planning systems. The 3D-CRT, _S_IMRT, and VMAT plans were created with the RayStation (RayStation 5.0; RaySearch Laboratories, Stockholm, Sweden), and tomotherapy plans were generated with a TomoTherapy Hi-Art System (Accuray Inc., Madison, WI, USA). The 3D-CRT plans comprised four 10-MV energy beams, arranged as anterior-posterior opposed beams and two lateral beams. The _S_IMRT plans were created using 7 angles (0°, 50°, 100°, 150°, 210°, 260°, and 315°) and a collimator angle of 90°, according to clinical experience. The VMAT plans used 2 full 6-MV arcs. The tomotherapy plans comprised helical beams optimized using a field width of 1.05°, modulation factor of 2.4, and pitch of 0.3. Each plan aimed to ensure 95% coverage of the PTV to the prescribed dose, with critical organ dose limits of 12.5 Gy and 10.0 Gy for the mean doses (D_mean_) to the liver and kidneys, respectively.

### Dosimetric parameters for plan evaluation

A radiation oncologist following the Radiation Therapy Oncology Group (RTOG) contouring atlases defined each OAR contour. Each OAR contour was contoured, and the body contour was automatically segmented using the “Whole Body” contouring tool with the MIM software (Cleveland, Ohio) in all patients. The dose distributions for each plan were analyzed using dose-volume histograms and dose distributions, and doses to the OARs were evaluated using the following criteria:

1. D_mean_ to each kidney, liver, and heart
2. Maximum dose (D_max_) to the bowel and spinal cord
3. D_mean_ to both lungs, volume of both lungs receiving ≥5 Gy (V_5_), and volume of both lungs receiving ≥20 Gy (V_20_)

The PTV doses were evaluated using the following parameters to evaluate target coverage and homogeneity:

1. Percent volume of the PTV receiving at least 95% of the prescription dose (TV_95_)
2. Homogeneity index (HI): HI = D_5_/D_95_

Where D_5_ and D_95_ represent the minimum doses to 5 and 95% of the PTV, respectively [19].

3) Conformity index (CI): CI = BV_95_/PTV

Where BV_95_ represents the volume of the body receiving 95% of the prescribed dose [20].

In our practice, we used a radiotherapy plan analysis program (Plan IQ™, Sun Nuclear co, Melbourne, FL, USA) to generate scores for these dosimetric goals according to the PQM. The scores were based on the constraints and dose-volume histograms of the planning results and assigned to each evaluation object according to the calculated dose [21, 22]. The quality scores for each objective are shown in Table 1. The PQM (%) represents the ratio of the raw PQM to the maximum PQM. The raw PQM is the evaluation score of each treatment plan in the score template. The maximum PQM is the highest score that the treatment plan can achieve and is the sum of the highest scores for each objective set by the user of plan IQ (set to 178). By using plan IQ, it is possible to create the score template for each objective that users want to evaluate. Depending on the treatment sites and prescribed dose, it is rated at a higher score compared to other scores for significant objectives. Because higher scores can be assigned to important OARs, dosimetric weighting is determined by clinical importance. To explain the score template, the score template for the kidney and lung D_mean_ (Gy) is shown in Additional file 1: Figure S1.

**Table 1:** Quality scores for each objective

	Target			Organ-at-risk (OAR)							
Score	PTV			Rt. kidney	Lt. kidney	Spinal cord	Liver	Heart	Lung		Bowel^a^
	D_95_ (cGy)	HI	CI	D_mean_ (cGy)	D_mean_ (cGy)	D_max_ (cGy)	D_mean_ (cGy)	D_mean_ (cGy)	D_mean_ (cGy)	V20 (cm^3^)	D_max_ (cGy)
0	< 2550	1.0	–	> 1800	> 1800	> 3000	≥2000	> 1000	≥900	> 2000	
1	2550	0.9	< 0.2, > 1.8	1800	1800	3000	1950	1000	950	2000	
2	2600	0.8	0.2, 1.8	1700	1700	2900	1900	950	900	1900	
3	2650	0.7	0.3, 1.7	1600	1600	2800	1850	900	850	1800	
4	2700	0.6	0.4, 1.6	1500	1500	2700	1800	850	800	1700	
5	2750	0.5	0.5, 1.5	1400	1400	2600	1750	800	750	1600	
6	2800	0.4	0.6, 1.4	1300	1300	2500	1700	750	700	1500	2700
7	2850	0.3	0.7, 1.3	1200	1200	2400	1650	700	650	1400	2750
8	2900	0.2	0.8, 1.2	1100	1100	2300	1600	650	600	1300	2800
9	2950	0.1	0.9, 1.1	1000	1000	2200	1550	600	550	1200	2850–2950
											3050–3150
10	3000	0.0	1.0	950	950	2100	1500	550	500	1100	3000
11				900	900	2000	1450	500	450	1000	
12				850	850	1900	1400	450	400	900	
13				800	800	1800	1350	400	350	800	
14				750	750	1700	1300	350	300	700	
15				700	700	1600	1250	300	250	600	
16				650	650	1500	1200	250	200	500	
17				600	600	1400	1150	200	150	400	
18				550	550	1300	1100	150	100	300	
19				500	500	1200	1050	100		200	
20				400	400	1100	1000	50		100	

The sum of scores for each objective is defined as the “raw plan quality metric (PQM)”. The maximum score was set to 178, and the PQM (%) was determined as the percent of the Raw PQM to Max PQM for each plan

^a^Bowel scoring was conducted differently because part of the bowel is included in the PTV, and therefore, the bowel dose partly reflects the PTV coverage

*Abbreviations: PTV* planning target volume, *HI* homogeneity index, *CI* conformity index

### Statistical analysis

The Wilcoxon signed-rank test and Friedman test were used for the group-wise statistical comparison of the 8 planning techniques (3D-DIBH, 3D-FB, _S_IMRT-DIBH, _S_IMRT-FB, VMAT-DIBH, VMAT-FB, Tomo-DIBH, and Tomo-FB). A *p*-value of < 0.05 was considered statistically significant. Statistical analyses were performed using SPSS software (Ver. 23.0; SPSS Inc., Chicago, IL, USA).

## Results

### Target coverage, conformity, and homogeneity

**Fig. 1:**
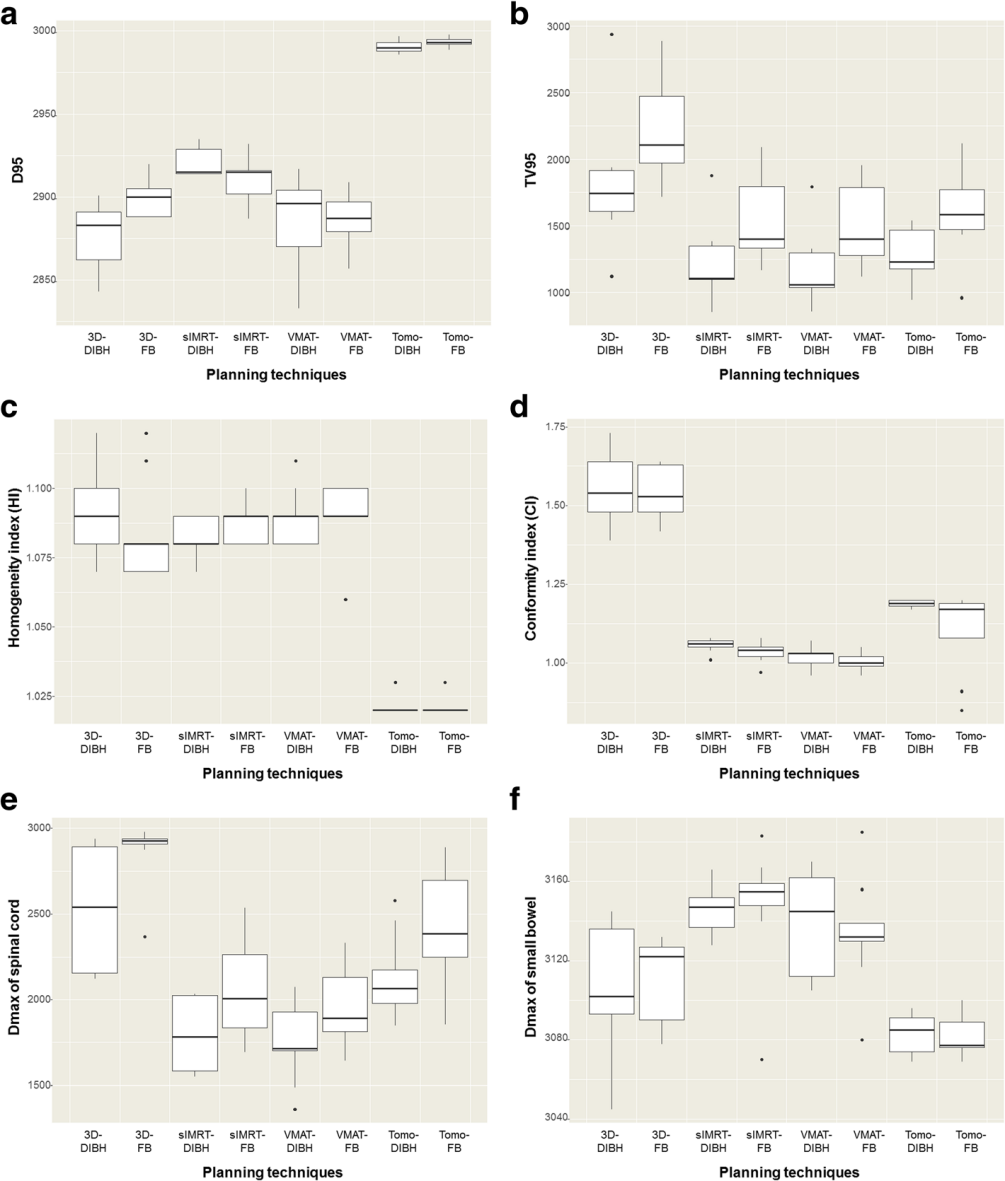
Boxplots of (**a**) the doses to 95% of the PTV (D_95_), (**b**) percent volume of the PTV receiving at least 95% of the prescription dose (TV_95_), (**c**) homogeneity index (HI), (**d**) conformity index (CI), (**e**) D_max_ of the spinal cord, and (**f**) D_max_ of the small bowel of the 8 different plan groups. *The significant differences between groups are shown in the Supplementary text

All plans satisfied the criteria for good PTV coverage, conformity, and homogeneity. Accordingly, the group-wise comparison of PTV dose distribution involved the TV_95_ and D_95_ values of the 8 techniques. Although all D_95_ values were larger than 95% of the prescribed dose, the tomotherapy plans yielded the best result, followed by _S_IMRT, VMAT, and 3D-plans, with no differences between the DIBH and FB plans. Statistically, the IMRT plans were significantly better than 3D-CRT plans, _S_IMRT plans were significantly better than VMAT plans, and tomotherapy plans were significantly better than _S_IMRT plans. The mean D_95_ values of the 3D-DIBH, 3D-FB, _S_IMRT-DIBH, _S_IMRT-FB, VMAT-DIBH, VMAT-FB, Tomo-DIBH, and Tomo-FB plans are shown in Table 2 and Fig. 1(a) (*p* < 0.001). The detailed results of statistical analyses are presented in Additional file 2: Supplementary text 1.

**Table 2:** Comparison of the 8 plans (mean ± standard deviation of 9 patients)

	Variables (mean ± SD)												
Plan	TV95 (cc)	D95 (cGy)	HI	CI	Rt. Kidney D_mean_ (cGy)	Lt. kidney D_mean_ (cGy)	Spinal cord D_max_ (cGy)	Liver D_mean_ (cGy)	Heart D_mean_ (cGy)	Lung D_mean_ (cGy)	Bowel D_max_ (cGy)	PQM (%)	MU
All	1546.53 ± 55.12	2919.06 ± 5.72	1.07 ± 0.00	1.19 ± 0.03	665 ± 36	743 ± 56	2194 ± 52	1482 ± 24	559 ± 31	331 ± 10	3118 ± 4	66.2 ± 1.2	2110.13 ± 382.92
3D-DIBH	1810.27 ± 162.59	2881.44 ± 9.95	1.09 ± 0.01	1.55 ± 0.04	741 ± 139	847 ± 219	2545 ± 117	1558 ± 50	446 ± 46	321 ± 22	3103 ± 12	60.6 ± 2.4	178.11 ± 2.03
3D-FB	2257.54 ± 136.08	2890.44 ± 9.56	1.08 ± 0.01	1.54 ± 0.03	845 ± 141	1069 ± 217	2869 ± 64	1715 ± 42	854 ± 84	404 ± 33	3110 ± 7	50.9 ± 2.1	176.13 ± 1.55
sIMRT-DIBH	1230.34 ± 100.57	2914.89 ± 7.36	1.08 ± 0.00	1.06 ± 0.01	602 ± 80	551 ± 105	1787 ± 70	1288 ± 41	363 ± 41	281 ± 16	3146 ± 4	75.6 ± 1.7	355.91 ± 12.74
sIMRT-FB	1525.70 ± 106.21	2906.00 ± 7.17	1.09 ± 0.00	1.03 ± 0.01	697 ± 93	716 ± 109	2061 ± 91	1426 ± 33	697 ± 82	344 ± 27	3148 ± 11	66.6 ± 2.2	385.92 ± 20.04
VMAT-DIBH	1178.59 ± 95.73	2886.67 ± 9.19	1.09 ± 0.00	1.02 ± 0.01	504 ± 79	515 ± 100	1756 ± 80	1264 ± 31	342 ± 47	260 ± 18	3138 ± 9	76.8 ± 2.0	310.89 ± 10.56
VMAT-FB	1478.46 ± 103.17	2890.22 ± 9.37	1.09 ± 0.00	1.00 ± 0.01	618 ± 80	612 ± 101	1979 ± 81	1391 ± 47	683 ± 82	325 ± 27	3134 ± 9	69.3 ± 1.9	339.82 ± 12.33
Tomo-DIBH	1269.43 ± 64.38	2989.44 ± 1.990	1.02 ± 0.00	1.19 ± 0.00	598 ± 75	755 ± 156	2134 ± 81	1565 ± 53	380 ± 39	330 ± 20	3083 ± 3	69.2 ± 2.3	7567.89 ± 451.12
Tomo-FB	1621.90 ± 114.87	2993.33 ± 0.93	1.02 ± 0.00	1.10 ± 0.04	717 ± 93	879 ± 180	2418 ± 108	1652 ± 59	706 ± 70	381 ± 28	3082 ± 4	62.1 ± 2.8	7566.33 ± 524.30
*p* value*	< 0.001	< 0.001	< 0.001	< 0.001	0.575	0.460	< 0.001	< 0.001	< 0.001	0.002	< 0.001	< 0.001	< 0.001

Data are presented as means over the 9 investigated patients, and errors indicate inter-patient variability at the level of 1 standard deviation. ^*^P values came from the comparisons among 8 plans by each dosimetric parameter

*Abbreviations: SD* standard deviation, *DIBH* deep-inspiration breath hold, *FB*, free-breathing, *IMRT* intensity-modulated radiotherapy, *VMAT* volumetric-modulated arc therapy, *TV* the volume of the body receiving 95% of the prescribed dose, *HI*, homogeneity index; *CI* conformity index, *PQM* plan quality metric, *MU* motor unit

Regarding the TV_95_, significantly larger irradiated volumes were observed with FB plans than with DIBH plans (mean: 1721 cc for FB > 1372 cc for DIBH, *p* < 0.001), indicating the need to compensate for respiratory movement. The mean TV_95_ values of the 3D-DIBH, 3D-FB, _S_IMRT-DIBH, _S_IMRT-FB, VMAT-DIBH, VMAT-FB, Tomo-DIBH, and Tomo-FB plans are shown in Table 2 and Fig. 1(b) (p < 0.001). On statistical analyses, 3D plans yielded significantly higher TV_95_ values than IMRT plans, whereas VMAT plans had the lowest TV_95_ when plans using the same respiration modality were compared. The results of statistical analyses are presented in Additional file 2: Supplementary text 2.

Regarding homogeneity, the 3D, _S_IMRT, and VMAT plans yielded similar HI values, regardless of respiratory movement. Although plans showed favorable homogeneity, the tomotherapy plans were the most superior, regardless of respiratory movement. The mean HI values of the 3D-DIBH, 3D-FB, _S_IMRT-DIBH, _S_IMRT-FB, VMAT-DIBH, VMAT-FB, Tomo-DIBH, and Tomo-FB plans are shown in Table 2 and Fig. 1(c) (*p* < 0.001). The detailed results of statistical analyses are presented in Additional file 2: Supplementary text 3.

**Fig. 2:**
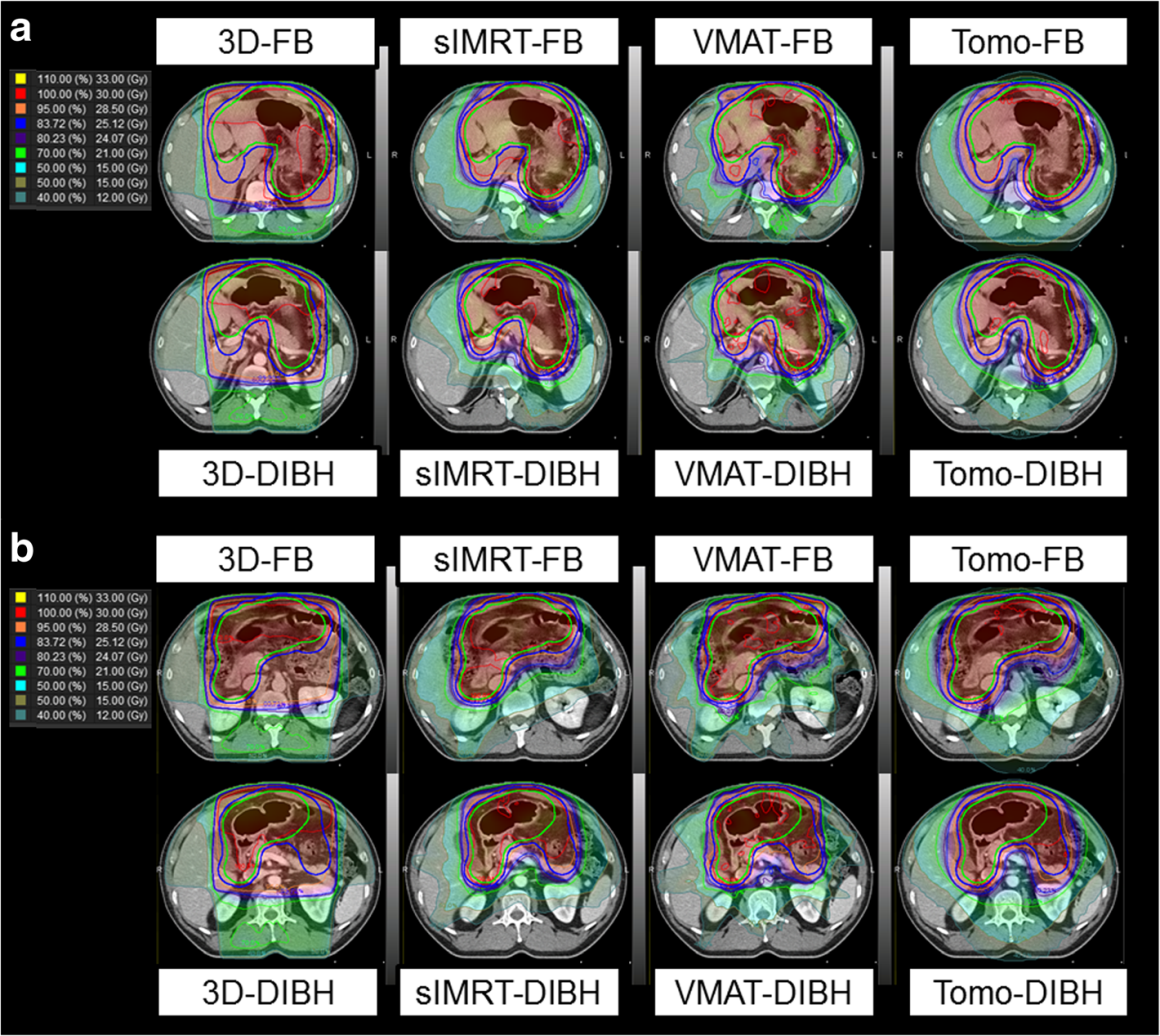
Treatment plans for 1 patient; (**a**) isodose lines in the image showing the curvature of the stomach, (**b**) isodose lines in the image showing the irradiation dose to both the kidneys. In Fig. 2(a), the difference in D_95_, TV_95_, HI, and CI values among treatment plans can be compared visually to some extent. In Fig. 2(b), the difference between the right and left kidney D_mean_, liver D_mean_, and bowel D_max_ doses among treatment plans can be compared visually to some extent

Regarding dose conformity, the 3D plans yielded the worst CI values, followed by IMRT plans (which had similar CI values). Among the IMRT plans, _S_IMRT and VMAT yielded the best CI values. As shown in Fig. 2, which demonstrates the isodose lines, 3D-DIBH and 3D-FB were the least conformal plans, followed by Tomo-DIBH and Tomo-FB. No differences were observed according to respiratory movement. The mean CI values of the 3D-DIBH, 3D-FB, _S_IMRT-DIBH, _S_IMRT-FB, VMAT-DIBH, VMAT-FB, Tomo-DIBH, and Tomo-FB plans are shown in Table 2 and Fig. 1(d) (*p* < 0.001). The detailed results of statistical analyses are presented in Additional file 2: Supplementary text 4, and differences in homogeneity and conformity among the plans are demonstrated in an example of the plans with isodose lines (Fig. 2).

### OAR doses

The DIBH plans, which involved smaller irradiated volumes, yielded lower kidney D_mean_ values on both sides relative to the FB plans. The D_mean_ values were the highest for the 3D-CRT plans, followed by tomotherapy, _S_IMRT, and VMAT, although these differences were not significant. Similarly, the DIBH plans yielded significantly lower D_mean_ values for the liver, heart, and lungs, compared to FB plans. For these OARs, the VMAT-DIBH and _S_IMRT-DIBH plans were significantly superior to the others, whereas the Tomo-FB and 3D-FB plans yielded the worst outcomes. As shown in Fig. 2, the DIBH plans yielded smaller irradiated volumes for the liver and kidneys, compared to the corresponding FB plans.

For the spinal cord, DIBH plans yielded significantly lower D_max_ values, compared to FB plans. 3D-CRT plans yielded the highest values, followed by tomotherapy, _S_IMRT and VMAT plans. The values and results of statistical analyses are presented in Fig. 1(e) and Additional file 2: Supplementary text 5. In contrast, the D_max_ values for the small bowel did not differ significantly among the plans (Fig. 1(f)), although the _S_IMRT plans and VMAT plans yielded the highest values, followed by the 3D plans and Tomo plans. Respiratory movement also had no effect on the small bowel D_max_ values. The results of statistical analyses are presented in Additional file 2: Supplementary text 6, and the mean values for each OAR with each plan are shown in Table 2.

### PQM

**Fig. 3:**
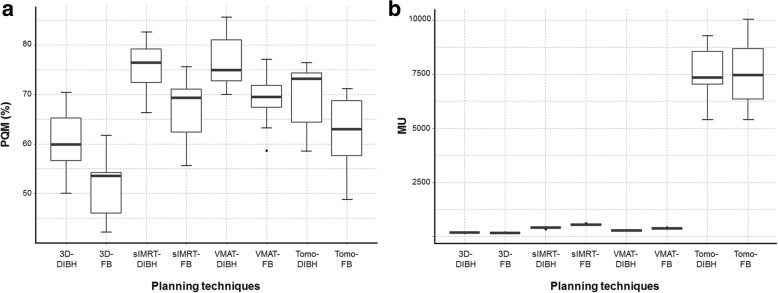
Boxplots of (**a**) plan quality metric (PQM) scores and (**b**) motor units (MU) of the 8 different plan groups. DIBH, deep-inspiration breath hold; FB, free-breathing; 3D, 3-dimensional conformal radiotherapy; IMRT, intensity-modulated radiotherapy; VMAT, volumetric-modulated arc therapy; Tomo, tomotherapy

As shown in Table 1, the PQM score (%) of each plan was determined after weighting each dosimetric factor according to clinical importance. The VMAT-DIBH plan and _S_IMRT-DIBH plan acquired the best mean PQM scores (%) of 76.8 and 75.6, respectively. The VMAT-FB, Tomo-DIBH, _S_IMRT-FB, and Tomo-FB plans acquired mean PQM scores of 69.3, 69.2, 66.6, and 62.1, respectively. Both 3D-DIBH and FB plans acquired the lowest PQM scores (%) of 60.6 and 50.9, respectively (Table 2). All DIBH plans yielded significantly superior PQM scores to the corresponding FB plans (Fig. 3(a)). Although VMAT-DIBH and _S_IMRT-DIBH were significantly better than most other plans, these two plans were not significantly different (*p* = 0.066). The VMAT-FB and _S_IMRT-FB plans were significantly better than 3D-CRT plans (DIBH or FB), but significantly worse than those obtained using the DIBH technique (VMAT-DIBH or _S_IMRT-DIBH). When we further scored the plans by summing all items at a 1:1 ratio (not weighting each dosimetric factor according to clinical importance), the same plan quality rankings as PQM (weighting each dosimetric factor by users according to clinical importance) were achieved. As shown in Table 3, the VMAT-DIBH, _S_IMRT-DIBH, and Tomo-DIBH plans yielded the best results, and all were superior to the corresponding FB plans. The 3D-DIBH and 3D-FB plans yielded the most inferior scores for almost all factors.

**Table 3:** The ranking of each dosimetric variable (mean value) and scores generated by summing all rankings of the 8 plans

Variables	3D-DIBH	3D-FB	sIMRT-DIBH	sIMRT-FB	VMAT-DIBH	VMAT-FB	Tomo-DIBH	Tomo-FB
TV_95_	7	8	3	5	1	4	2	6
D_95_	7	4	3	4	6	5	2	1
HI	7	4	3	5	6	8	1	2
CI	8	7	4	3	2	1	6	5
Kidney D_mean_	7	8	3	5	1	4	2	6
Spinal cord D_max_	8	6	2	4	1	3	5	7
Liver D_mean_	5	8	2	4	1	3	6	7
Heart D_mean_	4	8	2	6	1	5	3	7
Lung D_mean_	3	8	2	6	1	4	5	7
Bowel D_max_	3	4	7	8	6	5	1	2
MU	2	1	5	6	3	4	8	7
Sum	61	66	36	56	29	46	41	57

*Abbreviations: DIBH* deep-inspiration breath hold, *FB* free-breathing, *IMRT* intensity-modulated radiotherapy, *VMAT* volumetric-modulated arc therapy, *TV* the volume of the body receiving 95% of the prescribed dose, *HI* homogeneity index, *CI* conformity index, *MU* motor unit

### Motor unit and clinical meaning

The IMRT plans yielded significantly higher monitor unit (MU) values, whereas the 3D-CRT plans yielded significantly lower MU values relative to the other modalities. Although the VMAT and _S_IMRT plans did not yield significantly different MU values, all were significantly lower than those of tomotherapy plans. The Tomo-DIBH and Tomo-FB plans required substantially high mean MU values (7567 and 7566, respectively; Table 2), and the MU values did not significantly differ between the DIBH and corresponding FB plans (Fig. 3(b)).

We note that when performing actual treatments using the DIBH technique, beam irradiation can be performed only when the patient is holding his/her breath (intervals of ~ 15 to 20 s). As free breathing must be allowed between the periods of breath holding, the total treatment time in an actual clinic setting may be longer than expected. In addition, patient training time, DIBH device set-up time, and accurate monitoring in the treatment room are required. Therefore, the time required for actual treatment could be significantly increased if the IMRT and DIBH techniques are combined.

## Discussion

In this study, modern radiotherapy plans combining IMRT and DIBH (VMAT-DIBH, _S_IMRT-DIBH) were found to be significantly superior to 3D plans for gastric MALToma. We further observed that tomotherapy plans had exceptionally high MU values and yielded no notable benefits in this context. Although it remains unclear whether DIBH or IMRT is more useful in a clinical setting, VMAT-FB seems to yield better dosimetric outcomes when compared to 3D-DIBH. One of the strengths of this study is that we performed a dosimetric comparison among various modern radiotherapy techniques combining DIBH and IMRT and suggested the most appropriate treatment combination strategy regarding target coverage, OAR doses, and comprehensive evaluation for gastric MALToma.

Since the Memorial Sloan-Kettering Cancer Center (MSKCC) first described the treatment of gastric MALToma with RT alone [3], subsequent studies have shown favorable results in various populations, including patients with *H. pylori*-independent disease [4–8]. However, significant variations in stomach size and shape, digestive movement, and respiratory motion are known to cause uncertainty during the simulation and delivery of treatment to the stomach, and a safe, efficient irradiation technique that can optimally overcome these uncertainties with adequate margins has not been well established. The simplest approach to this issue involves the addition of an adequate margin to the CTV. Previous studies of RT planning techniques for gastric lymphoma have recommended a PTV comprising the CTV plus a 1.5–2-cm margin in all directions [10, 12] for the delivery of radiation in a FB state. Other studies have used respiratory synchronized 4D-CT images to provide information about respiratory-induced organ motion during treatment planning and minimize motion uncertainties [13, 23]. Specifically, Matoma et al. [17] compared the usefulness of 4D-CT vs. a uniform margin for the treatment planning of gastric MALToma, and found that the former yielded a significantly smaller mean PTV volume, with significantly lower mean doses to the liver and heart. According to the International Lymphoma Radiation Oncology Group (ILROG) guidelines for the CTV [24], the entire stomach should be considered to harbor disease even if the tumor appears confined to one area, and abnormal or suggestive perigastric lymph nodes can be included in the CTV. Respiratory motion-induced changes in stomach position should be detected using 4D-CT simulation or fluoroscopy when determining the ITV, and an additional margin of approximately 1 cm is often added to the CTV for this purpose. The PTV (normally ~ 1 cm) should account for setup variations. Moreover, radiotherapy planning based on 4D-positron-emission tomography (PET)-CT/4D-CT together with online cone-beam CT might be helpful to define PTV margins used for optimizing individual target coverage and estimating interfractional or intrafractional gastric movement [25].

DIBH has become a standard cardiac-sparing technique during the treatment of left-sided breast cancer [26–29], and several experts in lymphoma treatment centers have recently reported the use of DIBH for mediastinal lymphoma [30–33]. In most patients, DIBH reduces heart and lung doses by elongating the heart, resulting in greater separation from the target volume and increasing the lung volume. In DIBH, smaller PTV margins could be applied to further accentuate the organ-sparing benefit. Therefore, this respiratory technique is being considered for use during the treatment of other organs (i.e., liver and stomach) at various specialized institutions, including ours. More recently, Wang et al. demonstrated that there could exist substantial interfractional variation in stomach volume despite treatment with breath-hold and restriction of oral intake, and daily CT image guidance RT (CT-IGRT), in combination with a DIBH, enabled better target coverage with even smaller PTV margins (0.5–1.0 cm) while treating gastric MALToma [34].

Several investigators have also described the use of IMRT for the treatment of mediastinal lymphoma [32, 35, 36]. As expected, IMRT improves target conformity and reduces OAR doses. However, this technique also increases the volume of low-dose exposure in tissues such as the lungs, heart, and breasts. Notably, the use of the IMRT or DIBH technique in the treatment of Hodgkin lymphoma results in better protection of the heart and lungs [30, 31, 37]. Therefore, the combination of IMRT and DIBH would be expected to further reduce the doses to the OARs. In addition to the information on the breath-hold technique included in the ILROG guidelines [38], more number of clinical attempts is ongoing to combine IMRT and DIBH effectively for Hodgkin lymphoma.

In contrast to the situation with Hodgkin lymphoma, only a few studies have evaluated the use of modern radiotherapy techniques for gastric MALToma. In the first planning study for gastric MALToma, conducted by the MSKCC to determine the most advantageous technique [10], the PTV was defined as the CTV plus a 2-cm margin to account for respiratory-induced movement of the stomach during FB. In a comparison of AP/PA, 3D-CRT, and IMRT plans, 4-field 3D-CRT markedly decreased the dose to the kidneys when this organ overlapped with the PTV, and the findings with IMRT plans suggested that the kidney and liver doses could be incrementally improved in selected patients. Two relevant studies were also reported by Korean researchers. Lim et al. [12] retrospectively compared 2 different planning techniques (2D and 3D-CRT) for gastric MALToma based on CT with a FB status; the PTV was defined as the CTV plus a 1–1.5-cm margin, and an additional 1-cm margin was added in the craniocaudal direction to compensate for respiratory-induced stomach motion. For 3D-CRT plans, AP/PA or 3–4 non-coplanar fields were used according to the physician’s preference. Although that retrospective study compared treatment outcomes rather than dosimetric planning, the 3D-CRT plans yielded significantly better PTV coverage, conformity, and kidney doses on both sides when compared to 2D-RT plans, without compromising the oncologic outcomes. Furthermore, Bae at al. [13] compared 5 planning techniques (AP/PA, 4-field, 3D-CRT, IMRT with only coplanar beams, and IMRT with a few non-coplanar beams). 4D-CT was conducted under a FB status, ITV was defined as the sum total of the entire stomach at every respiratory stage, and CTV was defined as the ITV plus a 1-cm margin. The authors observed the highest mean kidney and liver doses with the AP/PA plan and a 4-field plan, respectively. As observed in our study, Bae and colleagues observed better conformity and hepatic toxicity with IMRT plans, but found no significant difference between the coplanar and non-coplanar IMRT plans. Unlike our study, however, these previous studies were limited by a lack of comparison with the DIBH technique and with more recent IMRT plans (e.g., VMAT and tomotherapy). In our institution, we defined CTVs as the GTV plus a 1.5-cm margin for FB plans, and GTV plus a 1.0-cm margin for DIBH plans, without 4D-CT scans. There was only a small difference in CTV margins compared to that in other planning studies. Moreover, considering that this study was a dosimetric study for comparing optimal radiotherapy planning techniques in patients, the conclusion would not change and this seems reasonable compared to other studies.

Similar planning studies have been conducted abroad, although neither modern DIBH nor various IMRT planning techniques have been evaluated. A similar planning study that compared four-field 3D-CRT, half-field RT, and IMRT was performed by Japanese researchers [11]. In that study, planning CT was performed during shallow exhale and inhale phases; the CTVs from both phases were fused, peristalsis margins were added to obtain the ITV, and the PTV was generated by expanding the ITV by 1 cm in all directions. The IMRT and half-beam methods were found to reduce the doses to the kidneys and liver, compared to 3D-CRT. In a retrospective Chinese study [23], the dosimetric superiority and efficacy, toxicity, and quality of life were investigated in patients with gastric diffuse large B-cell lymphoma who received IMRT. IMRT was performed with a FB status, and the PTV was defined as the CTV plus 1–2 cm in all directions. The 5-year overall survival, progression-free survival, and locoregional control rates were 80, 75, and 93%, respectively, with excellent target coverage and long-term global and functional quality of life scores.

The application of DIBH to gastric lymphoma might differ significantly with respect to the dose and target location. The use of DIBH requires 10–20 min of coaching during the treatment simulation and prior to administration of the first fraction. A patient is expected to hold their breath for 10–20 s per respiratory cycle, during which radiation must be delivered. This limitation prolongs the daily treatment time by a few minutes, and the addition of IMRT can further affect the treatment time. Additionally, each treatment requires a very sophisticated protocol. However, patients with gastric lymphoma account for only a small proportion of departmental workloads, and therefore, these limitations may be outweighed by the improved treatment accuracy and reduction in late adverse events.

This study had a few limitations. First, the relatively small sample size might have limited our ability to make firm recommendations regarding the usefulness of DIBH. Second, as this was a treatment planning study, we could not demonstrate the oncologic outcomes or prevalence of secondary malignancies after long-term follow-up in the same cohort. Still, we note that almost 2 years have passed since our institution actually used this technique, and no treatment failures have been reported, although a long follow-up duration would be needed given the late disease recurrence of this disease entity. Third, the plan IQ-based scoring method only calculates the target and OAR doses using CT density, but does not consider the delivery modality. In fact, no precise radiotherapy plan scoring system uses subjective criteria to evaluate each objective item. In other words, it would not be possible to define an absolute score for each treatment plan, although relative comparisons among different plans are possible. Our results should be interpreted in consideration of the aforementioned points, and they need to be verified through further clinical studies.

## Conclusions

Our findings demonstrate that modern radiotherapy plans combining DIBH with VMAT or _S_IMRT were significantly more beneficial than 3D plans for gastric MALToma by saving the OARs and enhancing conformity, regardless of concerns about increased MUs. Although whether DIBH, VMAT, or _S_IMRT is more useful in the clinical setting is unclear, VMAT-FB seems to be better than 3D-DIBH regardless of target margins. Further studies are needed to confirm the relevance of the most practical plan considering clinical outcomes.

## Additional Files

Additional file 1: **Figure S1.** An example of the score template for the kidney and lung D_mean_ (Gy). Lower doses to the kidneys or lungs indicate better dosimetric distribution. Thus, a higher (superior) score can be acquired when the kidneys or lungs could be saved more, as the user set the template. (TIF 24 kb)

Additional file 2: **Supplementary text 1.** Significant results of statistical analyses using the Wilcoxon signed-rank test (D95). **Supplementary text 2.** Significant results of statistical analyses using the Wilcoxon signed-rank test (TV95). **Supplementary text 3.** Significant results of statistical analyses using the Wilcoxon signed-rank test (HI). **Supplementary text 4.** Significant results of statistical analyses using the Wilcoxon signed-rank test (CI). **Supplementary text 5.** Significant results of statistical analyses using the Wilcoxon signed-rank test (D_max_ of the spinal cord). **Supplementary text 6.** Significant results of statistical analyses using the Wilcoxon signed-rank test (D_max_ of the small bowel) (DOCX 16 kb)

## Abbreviations

3D-CRT: 3-dimensional conformal radiotherapy
AP/PA: Anterior-posterior/posterior-anterior fields
CT: Computed tomography
CTV: Clinical target volume
DIBH: Deep-inspiration breath hold
FB: Free-breathing
GTV: Gross tumor volume
IMRT: Intensity-modulated radiotherapy
ITV: Internal target volume
MALToma: Mucosa-associated lymphoid tissue lymphoma
MSKCC: Memorial Sloan-Kettering Cancer Center
MU: Monitor unit
NCCN: National Comprehensive Cancer Network
OAR: Organ-at-risk
PQM: Plan quality metric
PTV: Planning target volume
_S_IMRT: step-and-shoot IMRT
VMAT: Volumetric-modulated arc therapy
