# Supplementary figures and images for: Combining deep-inspiration breath hold and intensity-modulated radiotherapy for gastric mucosa-associated lymphoid tissue lymphoma: Dosimetric evaluation using comprehensive plan quality indices
Source: Radiat Oncol. 2019 Apr 8;14:59. doi: 10.1186/s13014-019-1263-7 (PMC6454700; doi:10.1186/s13014-019-1263-7)

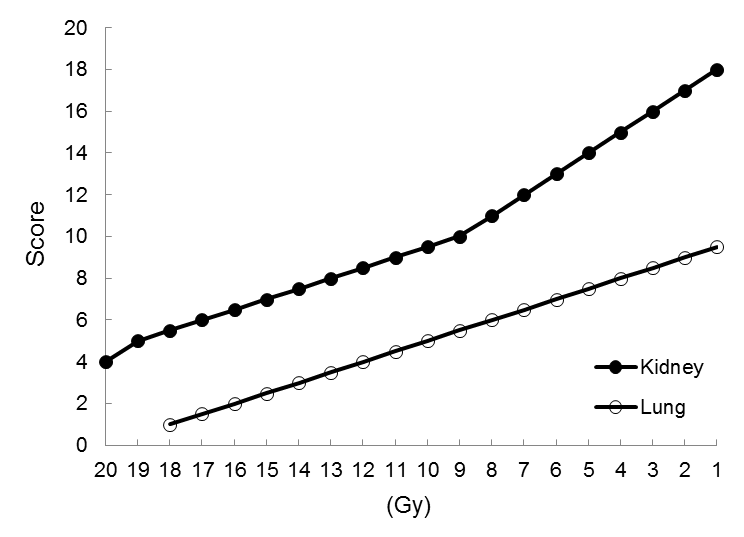

Supplement: Supplementary file 1 — Figure S1. An example of the score template for the kidney and lung Dmean (Gy). Lower doses to the kidneys or lungs indicate better dosimetric distribution. Thus, a higher (superior) score can be acquired when the kidneys or lungs could be saved more, as the user set the template. (TIF 24 kb) [file 13014_2019_1263_MOESM1_ESM.tif]
