# Supplementary material for: Combining deep-inspiration breath hold and intensity-modulated radiotherapy for gastric mucosa-associated lymphoid tissue lymphoma: Dosimetric evaluation using comprehensive plan quality indices
Source: Radiat Oncol. 2019 Apr 8;14:59. doi: 10.1186/s13014-019-1263-7 (PMC6454700; doi:10.1186/s13014-019-1263-7)
Supplement: Supplementary file 2 — Supplementary text 1. Significant results of statistical analyses using the Wilcoxon signed-rank test (D95). Supplementary text 2. Significant results of statistical analyses using the Wilcoxon signed-rank test (TV95). Supplementary text 3. Significant results of statistical analyses using the Wilcoxon signed-rank test (HI). Supplementary text 4. Significant results of statistical analyses using the Wilcoxon signed-rank test (CI). Supplementary text 5. Significant results of statistical analyses using the Wilcoxon signed-rank test (Dmax of the spinal cord). Supplementary text 6. Significant results of statistical analyses using the Wilcoxon signed-rank test (Dmax of the small bowel) (DOCX 16 kb) [file 13014_2019_1263_MOESM2_ESM.docx]

**Supplementary Material**

Supplementary text 1. Significant results of statistical analyses using the Wilcoxon signed-rank test (D95)

- **_S_**IMRT-DIBH, **_S_**IMRT-FB, Tomo-DIBH, or Tomo-FB >> 3D-DIBH (all p < 0.005)
- Tomo-DIBH or Tomo-FB >> 3D-FB (all p < 0.005)
- Tomo plans >> other IMRT or 3D plans (all p < 0.005)
- **_S_**IMRT-DIBH > VMAT-DIBH (p = 0.008)

Supplementary text 2. Significant results of statistical analyses using the Wilcoxon signed-rank test (TV95)

- 3D-FB >> all other plans (all p < 0.05)
- 3D-FB >> 3D-DIBH (p = 0.011)
- 3D-DIBH >> IMRT plans (**_S_**IMRT-DIBH, VMAT-DIBH, VMAT-FB, or Tomo-DIBH) (all p < 0.05)
- All FB plans > corresponding DIBH plans (all p < 0.05)
- **_S_**IMRT-FB >> Other IMRT-DIBH plans (**_S_**IMRT-DIBH, VMAT-DIBH, Tomo-DIBH) (all p < 0.05)
- Other IMRT-FB plans (**_S_**IMRT-FB, VMAT-FB, or Tomo-FB) >> VMAT-DIBH (all p < 0.05)
- Tomo-FB >> Tomo-DIBH (p = 0.019)

Supplementary text 3. Significant results of statistical analyses using the Wilcoxon signed-rank test (HI)

- Any other 3D or IMRT plans >> Tomo plans (all p < 0.05)

Supplementary text 4. Significant results of statistical analyses using the Wilcoxon signed-rank test (CI)

- 3D plans >> any other IMRT plans (all p < 0.05)
- **_S_**IMRT-DIBH > **_S_**IMRT-FB, VMAT-DIBH, VMAT-FB (all p < 0.05)
- Tomo-DIBH > any other IMRT or FB plans (all p < 0.05)
- Tomo-DIBH > Tomo-FB (p = 0.017)

Supplementary text 5. Significant results of statistical analyses using the Wilcoxon signed-rank test (D_max_ of the spinal cord)

- 3D-FB or DIBH plans >> any other IMRT plans (all p < 0.05)
- **_S_**IMRT-FB > **_S_**IMRT-DIBH (p = 0.011)
- VMAT-FB > VMAT-DIBH (p = 0.013)
- Tomo-FB > Tomo-DIBH (p = 0.008)
- Tomo-FB > **_S_**IMRT or VMAT plans (all p < 0.05)
- No significant difference between **_S_**IMRT-FB vs. VMAT-FB or between **_S_**IMRT-DIBH vs. VMAT-DIBH

Supplementary text 6. Significant results of statistical analyses using the Wilcoxon signed-rank test (D_max_ of the small bowel)

- 3D-DIBH << **_S_**IMRT-DIBH, **_S_**IMRT-FB (all p < 0.05)
- 3D-FB << **_S_**IMRT-DIBH and >> Tomo plans (all p < 0.05)
- Tomo plans << any other IMRT plans (all p < 0.05)
- Tomo-FB << Tomo-DIBH (p = 0.008)

Supplementary Fig. 1. An example of the score template for the kidney and lung D_mean_ (Gy)

Lower doses to the kidneys or lungs indicate better dosimetric distribution. Thus, a higher (superior) score can be acquired when the kidneys or lungs could be saved more, as the user set the template.
